# Supplementary material for: Impact of Postoperative Changes in Brain Anatomy on Target Volume Delineation for High-Grade Glioma
Source: Cancers (Basel). 2023 May 19;15(10):2840. doi: 10.3390/cancers15102840 (PMC10216722; doi:10.3390/cancers15102840)
Supplement: Supplementary file 1 [file cancers-15-02840-s001.zip › cancers-2383644-supplementary.pdf]

SUPPLEMENT

Supplement Table S1 Overview of individual patient and MRI characteristics (*n* = 28).
MRI = magnetic resonance imaging; f = female; m = male; gbm = glioblastoma; odg = oligodendroglioma; FLAIR = fluid-attenuated inversion recovery.
* There was no subdural haematoma in MRI 1 for this patient.

patient	sex	age (years)	interval MRI 1–2 (days)	diagnosis	tumour location	Δ resection cavity (cm^3)	Δ resection cavity (%)	Δ FLAIR (cm^3)	Δ FLAIR (%)	Δ resection cavity + FLAIR (cm^3)	Δ resection cavity + FLAIR (%)	Δ haematoma (mm)	Δ haematoma (%)
1	f	66	11	gbm	frontal	–4.1	–25.8%	–31.0	–42.9%	–20.0	–31.5%	+3.4	+60.7%
2	m	59	12	gbm	frontal	–3.9	–13.9%	–15.7	–18.5%	–32.0	–22.3%	–2.6	–23.9%
3	m	55	21	gbm	frontal	–20.2	–27.4%	–29.4	–17.4%	+5.7	+4.0%	–2.6	–100.0%
4	f	69	21	gbm	frontal	+8.5	+93.4%	–85.0	–59.4%	–94.9	–67.1%	–2.6	–30.2%
5	m	76	21	gbm	parietal	+17.6	+110.7%	+0.5	+0.2%	–30.1	–13.0%	–5.5	–50.5%
6	m	64	17	gbm	frontal	–5.1	–11.9%	–36.0	–23.8%	–106.5	–64.4%	–0.6	–5.1%
7	f	34	20	gbm	frontal	+10.3	+37.9%	–9.3	–10.8%	+18.7	+42.0%	+0.0	+0.0%
8	f	51	15	gbm	temporal	–9.2	–16.1%	–10.5	–7.9%	+4.3	+6.8%	–4.1	–33.6%
9	m	50	35	gbm	occipital	–0.6	–9.8%	–23.4	–51.4%	–21.2	–46.9%	–0.6	–19.4%
10	m	52	50	odg	frontal	+3.4	+16.5%	–3.9	–6.6%	–31.4	–42.7%	–2.2	–48.9%
11	m	67	16	gbm	parietal	+67.8	+174.3%	–3.8	–1.4%	+8.1	+3.1%	+4.1	*
12	m	62	20	gbm	temporal	+1.6	+7.2%	–68.8	–55.6%	–69.6	–58.7%	–2.8	–65.1%
13	m	78	11	gbm	parietal	+12.0	+65.8%	–9.9	–15.5%	–21.4	–29.3%	+5.7	+101.8%
14	m	78	17	gbm	parietal	–2.7	–11.6%	–145.7	–75.4%	–42.1	–50.6%	–1.5	–11.1%
15	m	26	18	gbm	frontal	–18.6	–59.4%	–105.4	–72.0%	–24.9	–29.7%	–9.7	–56.7%

16	m	63	19	gbm	frontal	+13.4	+21.7%	–166.2	–62.9%	–70.6	–34.9%	–0.7	–9.3%
17	m	48	22	gbm	frontal	–7.5	–21.8%	–15.4	–14.9%	–5.3	–7.7%	–2.9	–36.7%
18	m	65	18	gbm	parietal	+1.7	+27.4%	–16.9	–39.0%	+2.9	+15.3%	–6.0	–44.8%
19	m	46	11	gbm	temporal	+7.0	+22.5%	–9.0	–14.8%	–3.5	–6.3%	+1.1	+14.9%
20	m	60	22	gbm	temporal	+7.1	+27.3%	–34.5	–43.5%	–25.3	–29.5%	–6.9	–39.9%
21	f	77	17	gbm	temporal	+11.6	+55.0%	–54.8	–49.4%	–41.3	–35.8%	–1.3	–9.8%
22	f	67	22	gbm	parietal	–11.5	–56.1%	–92.5	–60.6%	–92.1	–58.8%	–1.9	–13.8%
23	f	41	20	odg	frontal	–4.5	–20.8%	–6.1	–14.2%	–22.2	–32.7%	+1.8	+31.6%
24	m	70	8	gbm	frontal	–10.2	–26.8%	–89.3	–55.8%	–78.2	–53.7%	+5.3	+76.8%
25	f	53	14	gbm	frontal	–7.4	–13.7%	–75.7	–52.1%	–55.7	–35.3%	+0.0	+0.0%
26	f	60	28	gbm	parietal	–2.0	–23.5%	–92.7	–86.3%	–86.6	–88.5%	–4.1	–41.8%
27	m	73	22	gbm	temporal	–15.8	–30.5%	–69.0	–55.7%	–117.8	–64.5%	+5.0	+100.0%
28	f	59	26	gbm	temporal	–3.1	–37.1%	–14.8	–56.7%	–13.8	–63.6%	–3.5	–30.2%
